# Repair of Osteoporotic Bone Defects in Rats via the Sirtuin 1-Wnt/β-catenin Signaling Pathway by Novel Icariin/Porous Magnesium Alloy Scaffolds

**DOI:** 10.34133/bmr.0090

**Published:** 2024-12-09

**Authors:** Fei Yu, Geng Zhang, Jian Weng, Gaozhi Jia, Chongzhou Fang, Huihui Xu, Ao Xiong, Haotian Qin, Tiantian Qi, Qi Yang, Guangyin Yuan, Hui Zeng, Yuanchao Zhu

**Affiliations:** ^1^ Department of Bone & Joint Surgery, Peking University Shenzhen Hospital, Shenzhen Peking University-The Hong Kong University of Science and Technology Medical Center, 518036 Shenzhen, Guangdong, China.; ^2^ National & Local Joint Engineering Research Center of Orthopaedic Biomaterials, Peking University Shenzhen Hospital, Shenzhen 518036, China.; ^3^ Shenzhen Key Laboratory of Orthopaedic Diseases and Biomaterials Research, Shenzhen 518036, China.; ^4^ School of Intelligent Manufacturing and Equipment, Shenzhen Institute of Information Technology, Shenzhen 518172, China.; ^5^ Peking University Shenzhen Hospital, Shenzhen 518036, China.; ^6^ Department of Medical Ultrasound, Peking University Shenzhen Hospital, Shenzhen 518036, China.; ^7^ Shanghai Jiao Tong University, Shanghai 200240, China.; ^8^ Department of Orthopedics, Shenzhen Second People’s Hospital, Shenzhen 518035, China.

## Abstract

The slow rate of bone regeneration and repair in osteoporotic defects is one of the difficulties of clinical work. To prepare a novel icariin (ICA)/porous magnesium alloy scaffold and to investigate its effectiveness and possible mechanism in repairing osteoporotic bone defects, bilateral ovariectomy was performed on Sprague-Dawley rats. Then, a cylindrical bone defect was created in the model, and a novel ICA/porous magnesium alloy scaffold was prepared and implanted into the defect. Eight or 12 weeks after repairing, specimens and micro-computed tomography (CT) data were collected. Microscopic observation was fulfilled through hematoxylin and eosin, Goldner, Masson, periodic acid-Schiff, and Sirius red staining. The expression of proteins was detected by immunohistochemical staining. The novel ICA/porous magnesium alloy scaffold was noncytotoxic and biologically safe. After it was implanted into the defect for 8 or 12 weeks, the surface color and smoothness, depth, and area of the defect were better than those in the control group. Besides, there was sufficient osteoid tissue, more mineralized bones, more collagen fibers, and more polysaccharide components in the defect repaired with the ICA/porous magnesium alloy scaffold. These conditions are closer to those of real bones. Moreover, the repair effect improved with the repair time. Compared with those in the control group, the expression levels of Sirtuin 1(SIRT1), Wnt5a, β-catenin, glycogen synthase kinase 3β, alkaline phosphatase, runt-related transcription factor 2, bone morphogenetic protein-2, and osteocalcin proteins were elevated in bone tissue after the scaffold was implanted into the defect for 8 weeks (all *P* < 0.05). The novel ICA/porous magnesium alloy scaffold promotes the repair of osteoporotic bone defects in rats, a process that may be achieved through activation of the SIRT1-Wnt/β-catenin signaling pathway.

## Introduction

Osteoporosis (OP) is characterized by a reduction in the amount of bone tissue per unit volume, thinning of the bone cortex, a decrease in the number and size of spongy bone trabeculae, widening of the bone marrow cavity, and a declined load capacity, bone metabolic diseases that cause pain in the back and limbs, bone deformities, and even fractures [1]. The incidence of fractures in patients with OP is as high as 40%, with a 1-year mortality rate of 20% for osteoporotic fractures of the hip and spine, resulting in a marked economic burden [2]. As the life expectancy of the population increases, the probability of OP and its resulting serious complications also increases progressively [3]. Studies confirmed that when OP occurs, the ability of bone to heal and regenerate is weakened, and the resulting fragility fractures can cause delayed bone healing, bone nonunion, and even bone defects [4]. Clinical treatment of osteoporotic bone defects by bone transplantation is limited by the scarcity of donor bone sources and rendered ineffective by the immune rejection of allogeneic bone transplants and the lowered osteogenic capability of OP patients [5]. Therefore, artificial bone scaffolding biomaterials have been developed for the repair of osteoporotic bone defects.

Porous alloy scaffolds can promote cell adhesion and accelerate the regenerative repair process of bone and are widely used in bone tissue repair [6]. In recent years, magnesium biomaterials have attracted attention in the treatment of bone defects due to their excellent biosafety, biodegradability, biocompatibility, and osteoinductive properties [7]. Magnesium biomaterials have a similar modulus of elasticity to human bone, so unlike other materials, they avoid the “stress masking” effect after surgery [8]. Moreover, the magnesium ions produced by magnesium biomaterial degradation are involved in more than 300 enzymatic reactions in the body, contributing to protein synthesis and the maintenance of mitochondrial activity. Magnesium ions also play a crucial role in energy metabolism and maintaining cellular homeostasis [9]. In the field of orthopedics, magnesium ions promote osteogenic differentiation of bone marrow stromal stem cells (BMSCs) by regulating the function of the immune system and creating an immune microenvironment favorable for osteogenesis [10]. In addition, magnesium ions also encourage the secretion of calcitonin gene-related peptide during fractures, thereby accelerating fracture healing [11]. In the field of tissue engineering, researchers have created scaffolds containing magnesium ions through 3-dimensional (3D) printing and applied them to the repair of bone defects. Therefore, the construction of magnesium biomaterials as scaffolds for the treatment of osteoporotic bone defects will also lead to satisfactory results.

Originally recorded in the Shennong’s Herbal Classic, *Epimedium* is also known as a “Xianlingpi” herb in Chinese, capable of strengthening muscles and bones, tonifying the liver and kidneys, and dispelling wind and dampness. Icariin (ICA) is the main component of *Epimedium*, playing an important role in anti-inflammatory, antioxidant, antiapoptotic, and anti-OP activities [12]. ICA up-regulates the expression of runt-related transcription factor 2 (RUNX2) through the Wnt/β-catenin signaling pathway and thus promotes osteogenic differentiation of BMSCs [13]. ICA also stimulates bone formation and inhibits bone resorption through the transforming growth factor-β1/Smad pathway, a process in which the expression of bone morphogenetic protein-2 (BMP-2) is up-regulated [14]. Furthermore, ICA has been proved effective in accelerating fracture healing through the BMP-2/Smad5/Runx2 pathway when it is added to the daily diet of fractured rats [15]. The above findings provide a theoretical basis for the application of a novel ICA/porous magnesium alloy scaffold to the repair of osteoporotic bone defects in the present study.

The Sirtuin 1 (SIRT1) gene encodes a nicotinamide adenine dinucleotide-dependent lysine deacetylase [16], which can regulate the osteogenic differentiation, antioxidant, and adipogenesis of BMSCs through deacetylation [17]. SIRT1 can form a SIRT1-RUNX2 axis with the osteogenic marker RUNX2, thereby regulating bone metabolism and promoting bone formation [18]. In particular, the expression level of SIRT1 protein is significantly higher during OP treatment [19]. The Wnt/β-catenin signaling pathway is closely associated with the maintenance of homeostasis, organ development, the occurrence of multisystem diseases, and the physiopathological state of the skeleton. Wnt proteins bind to cell surface receptors to prevent β-catenin degradation and allow it to enter the nucleus and stimulate downstream gene expression [20]. There is a correlation between the SIRT1 gene and the Wnt/β-catenin signaling pathway [21]. They can act synergistically to regulate osteogenic differentiation and proliferation of BMSCs, thereby achieving the effects of OP treatment [22].

In the present study, we proposed the hypothesis that the novel ICA/porous magnesium alloy scaffold could promote the repair of osteoporotic bone defects through the SIRT1-Wnt/β-catenin signaling pathway. We prepared porous magnesium alloy scaffolds using hot-press sintering and loaded ICA onto the surface of the porous magnesium alloy scaffolds and implanted them into distal femoral bone defects in osteoporotic rats. This study of ours provide new ideas for the clinical treatment of osteoporotic bone defects.

## Materials and Methods

### Configuration of the BMSC culture medium and cell grouping

ICA and magnesium chloride were added separately to the complete medium of BMSCs (Cyagen, China). The complete media prepared contained different final concentrations of ICA (0, 10^−8^, 10^−7^, 10^−6^, 10^−5^, and 10^−4^ mol/l) and/or Mg^2+^ (0, 2, 4, 8, and 16 mmol/l). The 2 stimuli were divided into 30 groups in 2-by-2 combinations. Subsequently, the optimal concentrations of ICA, Mg^2+^, and ICA+Mg^2+^ for stimulating the proliferation of BMSCs in rats (Cyagen, China) were determined by the Cell Counting Kit-8 (CCK-8) test. Then, osteogenic induced differentiation media containing the optimal concentration(s) of ICA, Mg^2+^, or ICA+Mg^2+^ for rat BMSCs were prepared (Cyagen, China). Cells were divided into control group (no stimulation), ICA stimulation group (optimal concentration of ICA), Mg^2+^ stimulation group (optimal concentration of Mg^2+^), and ICA+Mg^2+^ group (optimal concentrations of ICA and Mg^2+^). The cells were incubated at 37 °C in a 5% CO_2_ incubator.

### CCK-8 test of BMSCs

BMSCs were cultured with a complete medium in a 96-well plate at a density of approximately 5 × 10^3^ cells per well. The cell medium (containing ICA and/or Mg^2+^ medium) was changed with a fresh medium after 1 and 4 d of cultivation. Each time after the medium was discarded, the cells were washed with phosphate-buffered saline (PBS), and 100 μl of the complete medium (containing ICA and/or Mg^2+^) and 10 μl of CCK-8 reagent were added to each well. The cells were then incubated for 60 min at 37 °C in a 5% CO_2_ incubator. The optical density (OD) per well was measured at a wavelength of 450 nm (Beckman Coulter DTX 880, Germany).

### β-Galactosidase staining of BMSCs

BMSCs were cultured with a complete medium in a 12-well plate at a density of approximately 1 × 10^4^ cells per well. The cell complete medium (containing ICA and/or Mg^2+^) was replaced with a fresh medium after 4 d of cultivation. Each time after the medium was discarded, the cells were washed with PBS, and 1 ml of β-galactosidase staining solution was added to each well. Then, the plate was incubated overnight at 37 °C in an incubator. Photographs were taken under a light microscope for observation.

### Cell scratch test of BMSCs

An appropriate amount of BMSCs were inoculated in a 12-well plate so that the fusion rate of the cells could reach 70%, and then the cells were cultured with a complete medium for 24 h. Subsequently, a vertical line was drawn with the tip of a 200-μl pipette in the center of the 12-well plate. The cells were then washed with PBS, and the complete medium (containing ICA and/or Mg^2+^) was replaced. Afterward, the plate was incubated at 37 °C in a 5% CO_2_ incubator. Photographs were taken at 0, 24, and 48 h with an inverted fluorescent microscope (Leica DMI8, Germany) for observation.

### Alkaline phosphatase staining of BMSCs

BMSCs were cultured with a complete medium in a 24-well plate at a density of approximately 3 × 10^5^ cells per well. After 24 h of incubation, the complete medium was replaced with the osteogenic induced differentiation medium (containing ICA and/or Mg^2+^). After 14 d of cultivation, the medium was discarded, and the cells were washed with PBS before being fixed in 4% paraformaldehyde for 20 min. Then, the cells were washed with PBS again. The alkaline phosphatase staining solution was prepared by adding appropriate amounts of 5-bromo-4-chloro-3-indolyl phosphate solution and nitro-blue tetrazolium chloride solution to the alkaline phosphatase (ALP) development buffer. The plate was incubated at room temperature against light for 24 h. Images were taken with a camera (Xiaomi 10s, China).

### Alizarin red-S staining of BMSCs

BMSCs were cultured with a complete medium in a 24-well plate at a density of approximately 5 × 10^5^ cells per well for 24 h. Then, the medium was replaced with the osteogenic induced differentiation medium (containing ICA and/or Mg^2+^). After 25 d of incubation, the medium was discarded, and the cells were washed with PBS prior to fixing in 4% paraformaldehyde for 20 min. The cells were then washed again with PBS. An appropriate amount of alizarin red-S staining solution was added to each well to stain the cells at room temperature for 30 min. After the cells were washed with PBS, images were taken with a camera (Xiaomi 10s, China).

### Detection of proliferation and osteogenic differentiation of BMSCs by reverse transcription quantitative polymerase chain reaction

BMSCs were cultured with a complete medium (containing ICA and/or Mg^2+^) for 4 d and the osteogenic induced differentiation medium (containing ICA and/or Mg^2+^) for 7 d. Cellular RNA was extracted using the RNA Extraction Kit (TIANGEN, China). Buffer RZ (1 ml) was added to each well, and supernatant was removed by centrifugation at 4 °C 12,000 rpm. Chloroform was then added, and the cells were centrifuged before being washed with Buffer RD. Afterward, the cells were recentrifuged and then washed with Buffer RW. NanoDrop 2000c (Thermo Fisher Scientific, Inc.) was used to measure the total RNA concentration. Reverse transcription synthesis of cDNA was performed using the HiScript III RT SuperMix for quantitative polymerase chain reaction (qPCR) following the instructions. The mRNA expression of target genes was detected on a Lightcycler 480 Real-Time PCR system (Roche Diagnostics GmbH, Mannheim, Germany) using the miScript SYBR Green PCR kit. The 20-μl reaction mix comprises 10 μl of 2X SYBR Green PCR Master Mix, 7.4 μl of ribonuclease-free water, 2 μl of cDNA template, 0.8 μl of 10X miScript Universal Primer, and 0.8 μl of specific mRNA primer. The number of reverse transcription qPCR (RT-qPCR) thermal cycling times was 40. The relative mRNA expression of target genes was analyzed by the relative standard curve method (2-ΔΔCT), with glyceraldehyde 3-phosphate dehydrogenase taken as the reference gene. The polymerase chain reaction primers used in this study are listed in Table S1.

### Western blot detection of osteogenic differentiation of BMSCs

BMSCs were cultured in the osteogenic induced differentiation medium (containing ICA and/or Mg^2+^) for 21 d. Then, total protein was extracted with radioimmunoprecipitation assay lysis buffer (Beyoncé, China), and the protein concentration was determined by a bicinchoninic acid protein analysis kit (Beyoncé, China). The proteins were treated by 10% sodium dodecylsulfate polyacrylamide gel electrophoresis so as to transfer them to a polyvinylidene fluoride membrane (Millipore, USA). The nonphosphorylated proteins were washed with tris-buffered saline with Tween 20 (TBST) containing 5% skim milk for 1 h, while the phosphorylated proteins was washed with TBST containing 5% bovine serum albumin for 1 h. The membrane was incubated overnight with primary antibodies (1:1,000), including anti-SIRT1 antibody (abcam, ab189494), anti-β-catenin antibody (CST, 8480), anti-p-β-catenin antibody (CST, 4176), anti-glycogen synthase kinase 3β (GSK3β) antibody (abclonal, A6164), anti-RUNX2 antibody (CST, 12556), anti-BMP-2 antibody (abclonal, A0231), and anti-β-actin (abclonal, AC026). After being washed in TBST, the membrane was incubated with secondary antibodies (1:5,000) (abclonal, AS014) for 1 h. The membrane was washed again in TBST, and luminescence reagent (biosharp, BL520B) was then added for developing and imaging (luminescence image analyzer, Amersham ImageQuant 800).

### Construction of the novel ICA/porous magnesium alloy scaffold

Porous magnesium alloy scaffolds are mainly composed of magnesium and also contain small amounts of Nd, Zn, and Zr (Mg–3.1 Nd–0.2 Zn–0.4 Zr, JDBM). The spherical NaCl particles were prepared as (3-mm-high) open porous scaffold templates of cylindrical shape through a hot-press sintering process. Briefly, at a pressure of 7.5 KPa, set the temperature increase rate to 2 °C/min for 24 h at 720 °C to obtain an open cell sodium chloride template. The liquid magnesium alloy was poured into the NaCl stencil at a speed of 3 mm/s and stopped at a maximum pressure of about 0.2 MPa, and then the NaCl stencil was removed by a flowing water bath. The solid was then subjected to ultrasonic cleaning, nitric acid alcohol etching, surface fluorination, and autoclaving to yield a complete porous magnesium alloy scaffold for retention. The scaffold was placed in 10^−6^ mol/l ICA anhydrous ethanol solution, shaken on a shaker for 24 h, and dried in a vacuum drying oven for 24 h. ICA was loaded onto the porous magnesium alloy scaffold by physical adsorption to construct the novel ICA/porous magnesium alloy scaffold.

### Scanning electron microscope observation of the novel ICA/porous magnesium alloy scaffold and magnesium ions release profile.

A scanning electron microscope (SEM) (TESCAN, Mira3 XMH) was used to characterize the novel ICA/porous magnesium alloy scaffold and the loading of ICA. All samples were gold sprayed prior to loading. Meanwhile, we used inductively coupled plasma optical emission spectrometry for the detection of magnesium ion release content in magnesium alloys and plotted the magnesium ion release curve. The dried porous magnesium alloy holder was put into PBS solution, and the PBS soaking solution was sampled at different time points, and new PBS was added. The samples were tested on the machine, and the parameters of inductively coupled plasma optical emission spectrometry were set as follows: radio frequency power: 1.50 KW, plasma flow: 15.0 l/min, Auxiliary flow: 1.00 l/min.

### Cytotoxicity and biosafety testing of the porous magnesium alloy scaffolds

The porous magnesium alloy scaffold leach liquor was prepared by soaking magnesium rods, which have the same composition as the porous magnesium alloy scaffold, in alpha modification of Eagle’s medium (α-MEM) for 3 d as described in ISO 10993-12-2021 at a rate of 0.1g/ml. The α-MEM leach liquor was diluted by 50% for subsequent experiments. BMSCs were spread at a density of 1 × 10^4^ cells per well in a 24-well plate with cell crawlers, and the cells were divided into α-MEM and α-MEM leach liquor groups. The corresponding medium was replaced every 24 h for 3 consecutive days. After the medium was removed, the cells were washed with PBS and fixed in 4% paraformaldehyde for 20 min. The cytoskeleton was washed with PBS containing 0.1% Triton X-100, stained with phalloidin for 50 min, washed in PBS containing 0.1% Triton X-100, and sealed with an antifade mounting medium with 4’,6-diamidino-2-phenylindole successively. Finally, the cytoskeleton was photographed under a confocal microscope (Leica, STELLARIS 5) for observation. After 1 d of culturing, the cells were fixed with 4% paraformaldehyde for 20 min, and the cell morphology was observed under an SEM. After 12 weeks of bone defect repairing, the heart, liver, spleen, lung, and kidney of Sprague-Dawley (SD) rats in the novel ICA/porous magnesium alloy scaffold group and the control group were collected, paraffin-embedded, and stained with hematoxylin and eosin (HE) for the analysis of the structural and cellular changes in the internal organs of the rats.

### Animals

Seven-week-old female SD rats (weight: 150 to 180 g) were purchased from the Experimental Animal Centre of Southern Medical University and housed in the general housing area of the animal laboratory at the Hospital Centre of Peking University Shenzhen Hospital in the following environmental conditions: 12-h-light/12-h-dark time each day, 50% to 55% relative humidity, and a constant room temperature of 24 °C. Rats had free access to the food and water they needed. The study was ethically approved by the Experimental Animal Management Committee of Shenzhen Hospital, Peking University, China (No. 2021-559). All experimental procedures were performed in accordance with the recommendations in the Institutional Animal Care Guide.

### Establishing and validating the OP model

The rats were placed in a gas anesthesia machine filled with isoflurane, and after successful anesthesia, each SD rat was subjected to bilateral ovariectomy (OVX). The surgical procedure was as follows: the rats’ bilateral lumbar dorsal fields were debrided and disinfected with iodine, and then the skin and muscle tissue were incised longitudinally on both sides of the spine to expose the abdominal cavity. The ovaries were searched retrograde along the fallopian tubes and finally found at the end of the tubes. The ovaries were removed, the stumps were ligated, and the incisions were closed in sequence and disinfected again (Fig. S1A). The rats were fed for 3 months to establish the OP model. Then, the micro-computed tomography (CT) scans of the lumbar vertebrae, femur, and tibia of rats in the normal group and the OVX group were taken. The results showed that rats in the OVX group had a thinner bone cortex, a reduced bone density, and a more sparse and disorganized distribution of trabeculae, compared with those in the normal group. Thus, we concluded that OP models were successfully established and could be used for subsequent experiments (Fig. S1B).

### Establishment of mouse models with osteoporotic bone defects and animal groups

The modeling procedure is shown in Fig. S1C. Specifically, the rats were first anaesthetized by isoflurane inhalation, and the right femur was incised at the lower outer end to expose the lateral femoral condyle, at the center of which a cylindrical bone defect of 3 mm in diameter and 3 mm in depth was made using a Kristen needle. The SD rats were randomly divided into 4 groups. In the Scaffold+ICA group (*n* = 16), the novel ICA/porous magnesium alloy scaffold was implanted in the bone defect of rats. In the Scaffold group (*n* = 16), a porous magnesium alloy scaffold was implanted in the bone defect. In the Control group (*n* = 16), no scaffold was implanted in the bone defect. In the Sham group (*n* = 48), only the lateral condyle of the left lower limb was exposed and sutured for sham surgery. All rats were subsequently housed for 8 or 12 weeks.

### Gross specimen observation

The rats were executed at corresponding time points. The ends of the femurs were cut bilaterally, and the area, depth, and color of the bone defect in the lateral femoral condyle were observed visually to assess bone repair.

### Micro-CT observation

The ends of the rat femur were scanned at corresponding time points using a Vivo 80 micro-CT (Scanco, Switzerland) at 70 kVp and 114 μA. 3D reconstruction was performed using Skyscan software.

### Evaluation of staining

Bone tissue samples were taken at corresponding time points at first and then fixed in 10% neutral buffered formalin solution for 48 h. After 6 weeks of decalcification in EDTA decalcifying solution, the samples were paraffin-embedded and then serially sectioned to 5-μm-thick pieces. The specific steps for HE staining included the following: hematoxylin staining for 3 min, water washing, alcohol fractionation with hydrochloric acid, eosin staining for 25 s, ethanol dehydration, and blocking. The specific steps for Goldner staining included: Weigert iron hematoxylin staining for 25 min, acid fractionation treatment for 5 s, acid Ponceau staining for 5 min, orange-G staining for 8 min, brilliant green staining for 5 min, ethanol dehydration, and blocking. The procedure for Masson staining was as follows: Weigert iron hematoxylin staining for 8 min, acid differentiation treatment for 5 s, Masson blue staining for 5 min, Lichon red magenta staining for 8 min, aniline blue staining for 1 min, ethanol dehydration, and blocking. The specific steps for periodic acid-Schiff staining included the following: Schiff staining for 15 min, water washing for 10 min, hematoxylin staining for 2 min, acid differentiation treatment for 5 s, dehydration, and blocking. The specific steps for Sirius red staining included the following: hematoxylin staining for 5 min, Sirius red staining for 24 h, hematoxylin staining for 2 min, acidic differentiation treatment for 5 s, film dehydration, and blocking. Photographs were taken under a microscope (OLYMPUS, BX53) for observation.

### Evaluation of immunohistochemistry

Bone tissue samples were taken at corresponding time points at first and then fixed in 10% neutral buffered formalin solution for 48 h. After 6 weeks of decalcification in EDTA decalcifying solution, the samples were paraffin-embedded and serially sectioned to 5-μm-thick pieces. The sections were immersed in pepsin for 25 min, washed with PBS, and then blocked with endogenous peroxidase for 10 min. The samples were washed again with PBS and blocked with nonspecific staining blockers for 10 min. After being washed with PBS, the samples were incubated overnight with primary antibodies (1:200) SIRT1, Wnt5a, β-catenin, GSK3β, ALP, RUNX2, BMP-2, and osteocalcin (OCN) (Bioss, Beijing, China). Afterward, the samples were treated by biotin-labeled sheep anti-mouse/rabbit immunoglobulin G polymer for 10 min, *Streptomyces* antibiotin protein-peroxidase for 10 min, and 3,3’-diaminobenzidine for color development successively. Finally, photographs were taken under a microscope (OLYMPUS, BX53) for observation.

### Statistical analysis

All data were expressed as x′ ± s. A *t* test was performed to compare the data between 2 groups of samples and a one-way analysis of variance was performed for multiple pairwise comparisons. SPSS 20.0 was used for statistical analysis, and ImageJ was employed for image analysis. A *P* value of <0.05 was considered statistically significant.

## Results

### Effect of costimulators ICA and Mg^2+^ on the proliferation of rat BMSCs

**Fig. 1.**
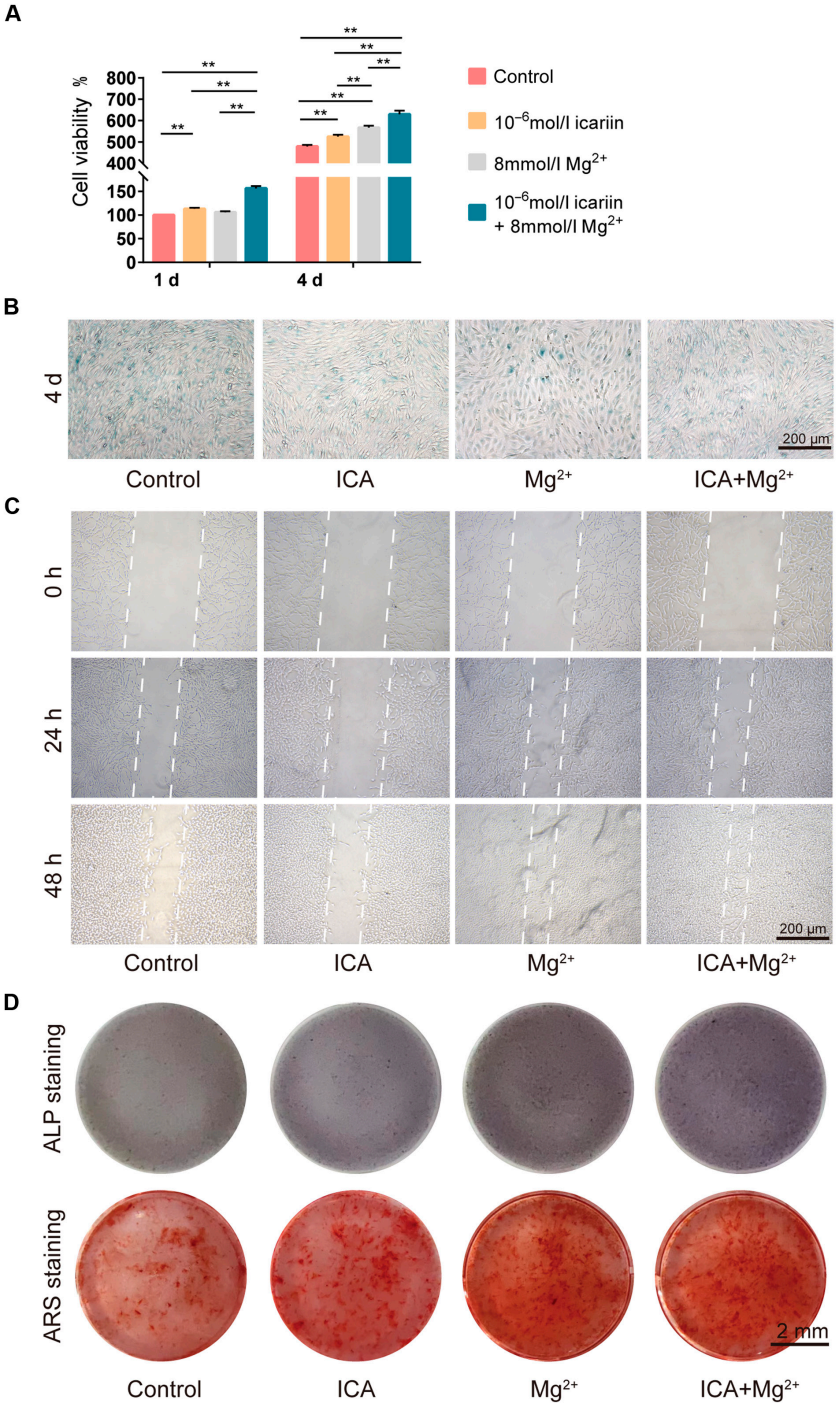
Experiments on proliferation, senescence, migration, and osteogenic differentiation of BMSCs. (A) OD values of BMSCs in the control group, ICA group, Mg^2+^ group, and ICA+Mg^2+^ group on the first day and fourth day of cultivation. (*, *P* < 0.05; **, *P* < 0.01). (B) β-Galactosidase staining of the effect of costimulators ICA and Mg^2+^ on the aging of BMSCs. (C) Microscopic observation of the effect of costimulators ICA and Mg^2+^ on the migration capacity of BMSCs. (D) ALP staining and alizarin red-S (ARS) staining for analyzing the effect of costimulators ICA and Mg^2+^ on the osteogenic differentiation of BMSCs.

The CCK-8 assay revealed that on the first day and fourth day of cultivation, the cells treated with 10^−6^ mol/l ICA had a higher OD value than those in the control group, but there was no statistical difference between these 2 groups (*P* > 0.05). On the fourth day, the cells treated with 8 mmol/l Mg^2+^ had a significantly higher OD value than those in the control group (*P* < 0.05). On the first day and fourth day, the cells treated with 10^−6^ mol/l ICA and 8 mmol/l Mg^2+^ had a significantly higher OD value than those in the control group (*P* < 0.05) (Fig. 1A and Table S2). Based on the above results, we divided the cells into 4 groups for subsequent experiments, namely, the control group, the ICA group (10^−6^ mol/l ICA), the Mg^2+^ group (8 mmol/l Mg^2+^), and the ICA+Mg^2+^ group (10^−6^ mol/l ICA+8 mmol/l Mg^2+^).

### Effect of costimulators ICA and Mg^2+^ on the aging of BMSCs

The aging of cells was observed by β-galactosidase staining on the fourth day of cultivation in different complete media. Compared with that in the control group, the number of aging cells decreased in the ICA group, Mg^2+^ group, and ICA+Mg^2+^ group. Among the 4 groups, the ICA+Mg^2+^ group had the fewest senescent cells (Fig. 1B).

### Effect of costimulators ICA and Mg^2+^ on the migration of BMSCs

On the first day and fourth day, the migration of cells cultivated in different complete media was observed by scratch tests. Compared to the control group, the ICA, Mg^2+^, and ICA+Mg^2+^ groups showed more intensified migration activities at 24 and 48 h. Among the 4 groups, the ICA+Mg^2+^ group had the strongest migration capacity (Fig. 1C).

### ICA and Mg^2+^ act synergistically to promote the mRNA expression of proliferation-related genes in rat BMSCs via the SIRT1-Wnt/β-catenin signaling pathway

**Fig. 2.**
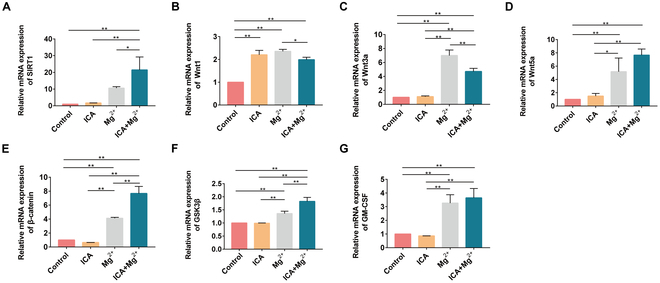
ICA and Mg^2+^ costimulate the mRNA expression of proliferation-related genes in rat BMSCs via the SIRT1-Wnt/β-catenin signaling pathway. (A) SIRT1. (B) Wnt1a. (C) Wnt3a. (D) Wnt5a. (E) β-catenin. (F) GSK3β. (G) GM-CSF. *, *P* < 0.05; **, *P* < 0.01.

The mRNA expression levels of SIRT1, Wnt1, Wnt3a, Wnt5a, β-catenin, GSK3β, and granulocyte-macrophage colony-stimulating factor (GM-CSF) were measured by RT-qPCR after 4 d of culturing in different complete media. The mRNA expression levels of SIRT1, Wnt1, Wnt3a, Wnt5a, β-catenin, GSK3β, and GM-CSF in the ICA+Mg^2+^ group were significantly higher than those in the control group (*P* < 0.05). The mRNA expression levels of Wnt1 and Wnt3a in the ICA+Mg^2+^ group were lower than those in the Mg^2+^ group (*P* < 0.05) (Fig. 2).

### Special staining for studying the effect of costimulators ICA and Mg^2+^ on the osteogenic differentiation of rat BMSCs

The osteogenic activity of BMSCs was observed by ALP staining and alizarin red-S staining after 14 and 25 d of cultivation in the osteogenic induction medium. After 14 d of culturing, ALP staining showed that ICA group stains darker compared to control group and the ICA+Mg^2+^ group had the greatest capacity for osteogenic differentiation (Fig. 1D). After 25 d of cultivation, alizarin red-S staining showed that ICA group staining has more red mineralized nodules compared to control, and the ICA+Mg^2+^ group had the strongest osteogenic differentiation capacity, with red-stained areas slightly darker than those of the Mg^2+^ group (Fig. 1D).

### ICA and Mg^2+^ costimulate the mRNA expression of genes related to osteogenic differentiation of rat BMSCs through the SIRT1-Wnt/β-catenin signaling pathway

**Fig. 3.**
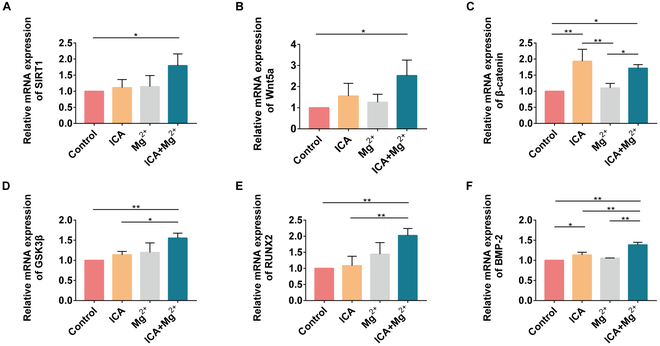
ICA and Mg^2+^ costimulate the mRNA expression of factors related to osteogenic differentiation of rat BMSCs via the SIRT1-Wnt/β-catenin signaling pathway. (A) SIRT1. (B) Wnt5a. (C) β-Catenin. (D) GSK3β. (E) RUNX-2. (F) BMP-2. *, *P* < 0.05; **, *P* < 0.01.

The expression levels of SIRT1, Wnt5a, β-catenin, GSK3β, RUNX-2, and BMP-2 mRNA were measured by RT-qPCR after 7 d of culturing in the osteogenic induction medium. The mRNA expression levels of SIRT1, Wnt5a, β-catenin, GSK3β, RUNX-2, and BMP-2 were significantly higher in the ICA+Mg^2+^ group than those in the control group (*P* < 0.05). The expression level of β-catenin was higher in the ICA group than that in the ICA+Mg^2+^ group, but the difference was not significant (*P* > 0.05) (Fig. 3).

### ICA and Mg^2+^ act synergistically to promote the expression of proteins related to osteogenic differentiation of BMSCs through the SIRT1-Wnt/β-catenin signaling pathway

**Fig. 4.**
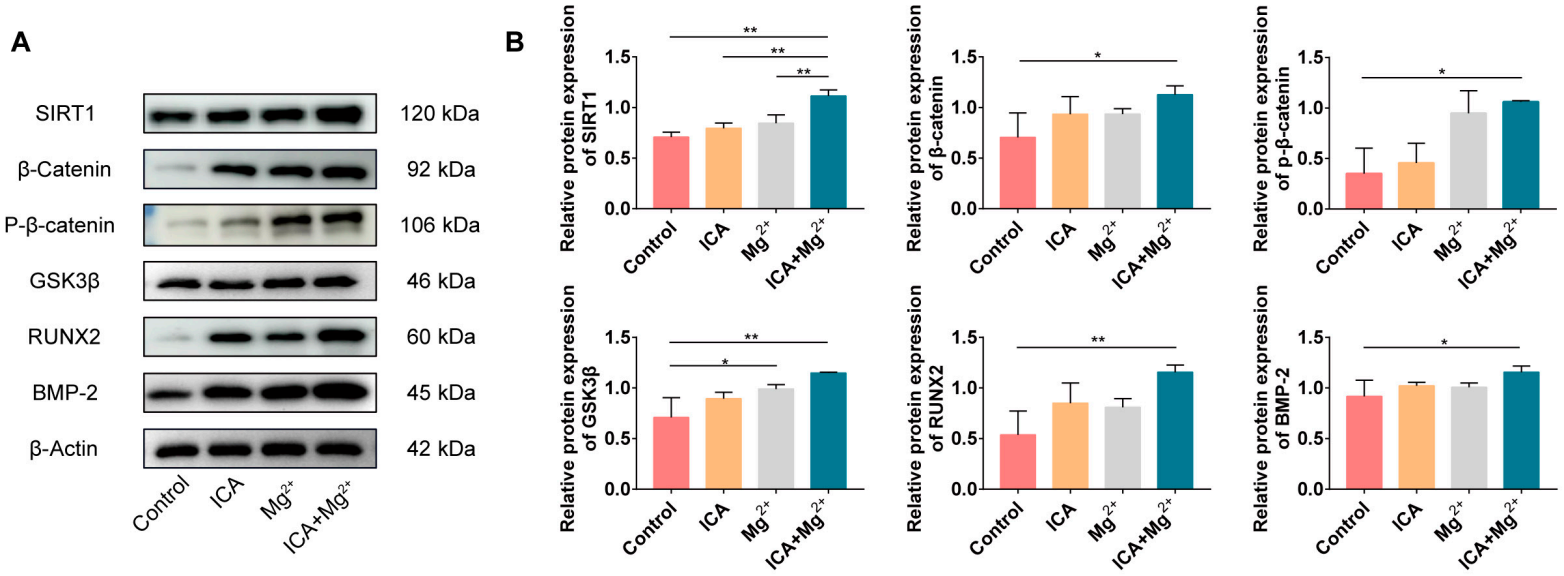
ICA and Mg^2+^ act synergistically to promote the expression of osteogenic differentiation-related proteins in rat BMSCs via the SIRT1-Wnt/β-catenin signaling pathway. (A) Results of Western blot experiment. (B) Statistical graph of Western blot experiment results. *, *P* < 0.05; **, *P* < 0.01.

The expression levels of SIRT1, Wnt5a, β-catenin, GSK3β, RUNX-2, and BMP-2 proteins were detected by Western blot after 21 d of cultivation in different osteogenic induction media. The expression levels of SIRT1, Wnt5a, β-catenin, p-β-catenin, GSK3β, RUNX-2, and BMP-2 proteins in the ICA+Mg^2+^ group were significantly higher than those in the control group (all *P* < 0.05) (Fig. 4).

### Morphological characterization of the novel ICA/porous magnesium alloy scaffold

**Fig. 5.**
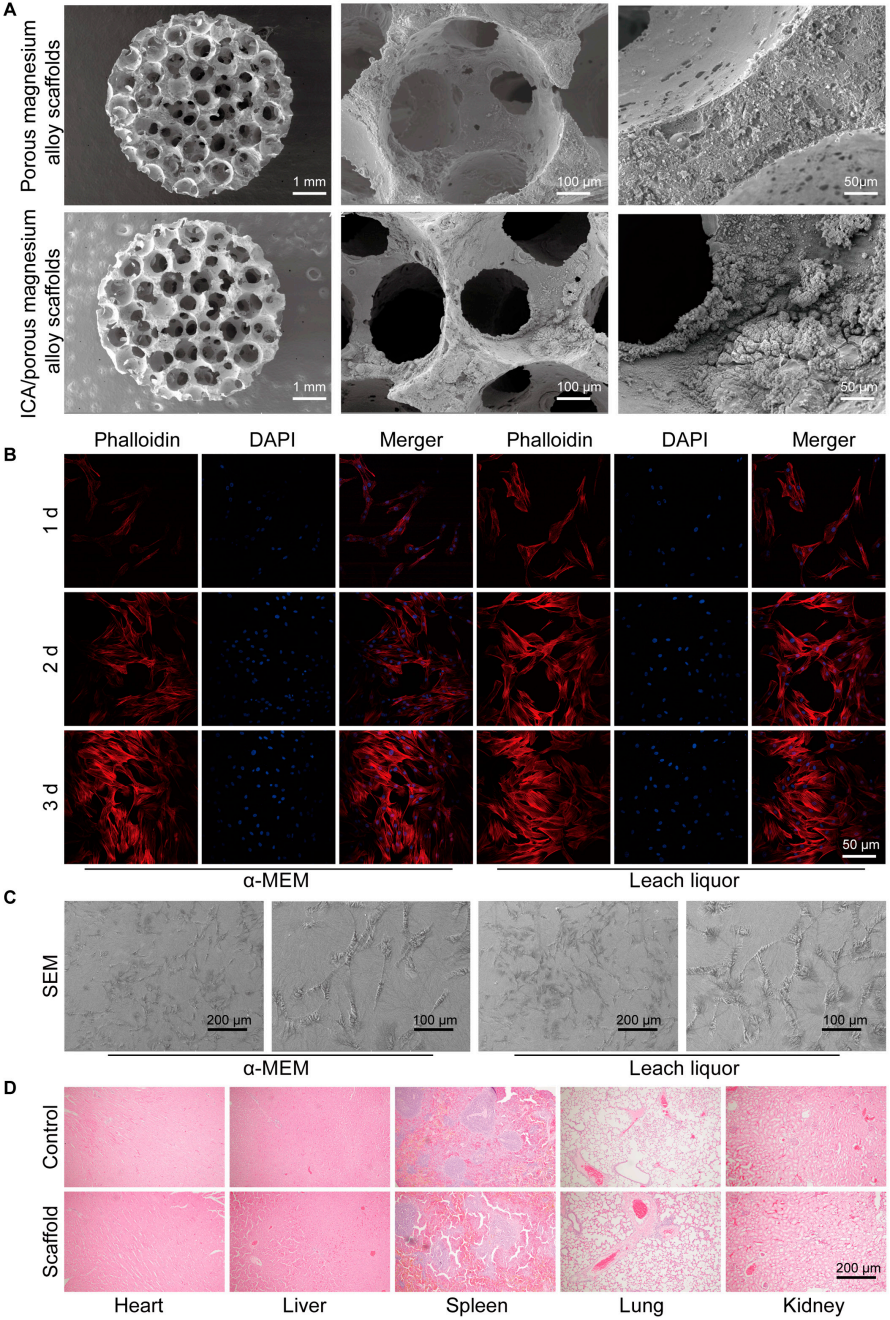
SEM and bulk sample observation of the surface structure of different scaffolds and biosafety of the porous magnesium alloy scaffold. (A) Porous magnesium alloy scaffold and novel ICA/porous magnesium alloy scaffold. (B) Confocal microscopic observation of BMSCs. (C) SEM observation of BMSCs and (D) visceral HE staining after implantation of the porous magnesium alloy scaffold in rats.

The novel ICA/porous magnesium alloy scaffold constructed had a cylindrical structure of 3 mm in diameter and 3 mm in height and macroscopic view of the porous magnesium alloy scaffold (Fig. S2). Surface structural characteristics of the porous magnesium alloy scaffold and the novel ICA/porous magnesium alloy scaffold were studied by an SEM. The porous magnesium alloy scaffold had a flatter surface with minor depressions on it, whereas the novel ICA/porous magnesium alloy scaffold had a rougher surface with precipitation on it. The precipitation was possibly ICA loaded on top of the porous magnesium alloy (Fig. 5A).

### Cytotoxicity and biosafety of porous magnesium alloy scaffolds and magnesium ions release profile in magnesium alloy scaffold

**Fig. 6.**
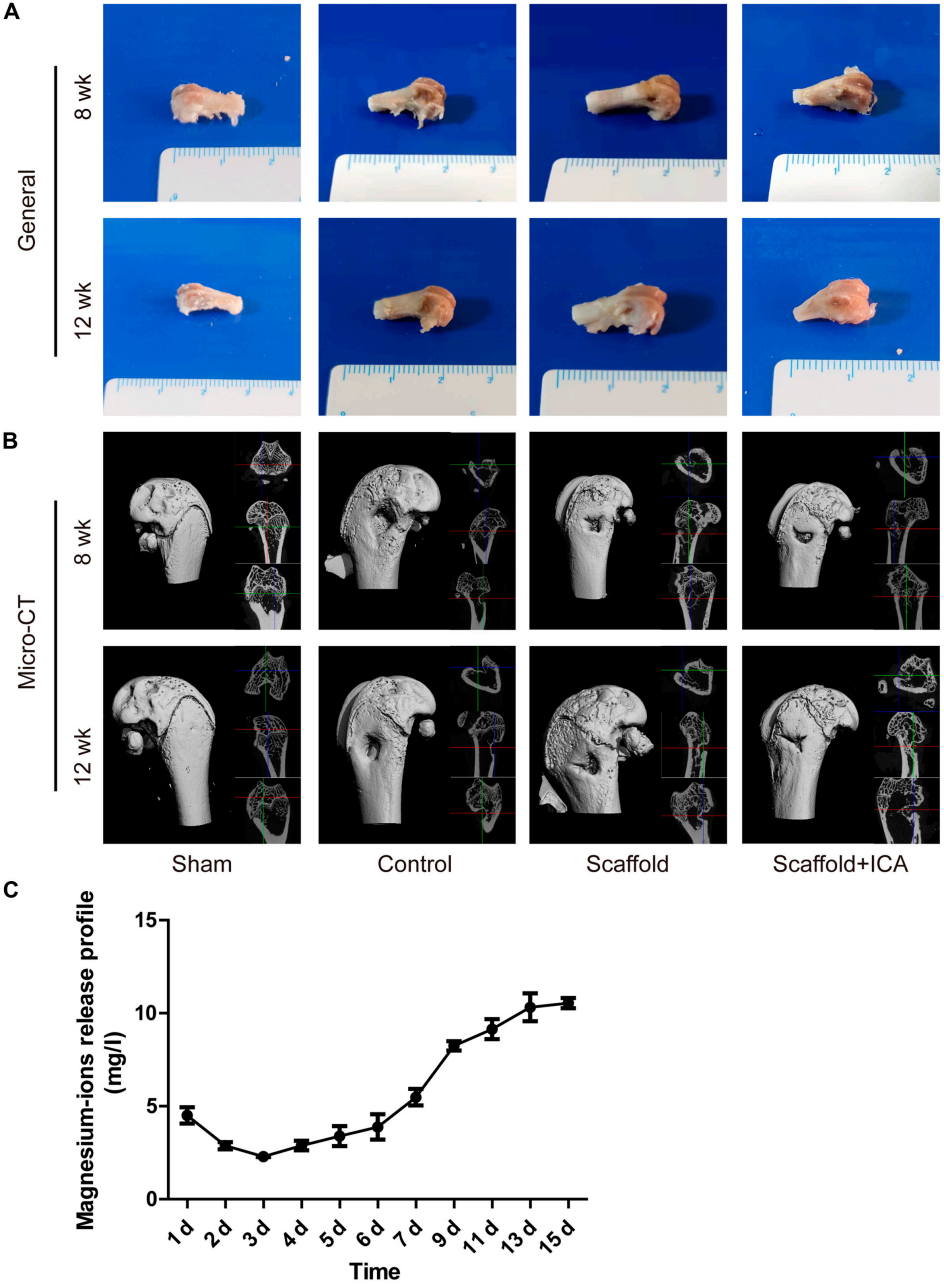
Macroscopic view of the specimen from osteoporotic bone defects repaired with different scaffolds. (A) Macroscopic view of the specimen. (B) Micro-CT reconstruction results. (C) Experimental magnesium ion release profiles of porous magnesium alloy scaffolds.

Confocal microscopy showed that 1 d after implantation, both groups of cells spread naturally, with increased pseudopods and a good distribution. The number of cells increased accordingly with the culturing days. Three days after implantation, the cells in the infusion group overlapped and had good integrity, and there was no cytoskeletal disruption (Fig. 5B). Electron microscopy showed that 1 d after implantation, the 2 groups of cells were morphologically similar with complete cell outlines (Fig. 5C). HE staining revealed that 12 weeks after implantation, the porous magnesium alloy implanted did not affect the fine structures of the heart, liver, spleen, lungs, and kidneys of the rats. The cell morphology was similar between the 2 groups, and no inflammatory cell infiltration occurred in both groups (Fig. 5D). The above findings proved that the porous magnesium scaffold was noncytotoxic and biologically safe. In the magnesium ion release profile experiment, the magnesium ion concentration was measured at 1, 2, 3, 4, 5, 6, 7, 9, 11, 13, and 15 d. The magnesium ion concentration at day 1 was 4.508 mg/l, which decreased to 2.296 mg/l at day 3. Thereafter, the concentration of magnesium ions released gradually increased, reaching 10.5498 mg/l at day 15 (Fig. 6C).

### Macroscopic specimens and micro-CT of samples from osteoporotic bone defects repaired with different scaffolds

After 8 weeks of repairing, the osteoporotic bone defect repaired with the ICA/porous magnesium alloy scaffold was more shallow and narrower than that in the other 2 groups. The micro-CT 3D reconstruction also revealed that the bone defect in the ICA/porous magnesium alloy scaffold group was repaired to the most extent, and more than adequate osteoid tissue filled inside. The bone defect was also well repaired in the porous magnesium alloy scaffold group, but the defect was still deeper than that in the ICA/porous magnesium alloy scaffold group. In the control group, a larger and deeper defect with a less smooth area was visible. After 12 weeks of repairing, the conditions of osteoporotic bone defects were better in all 3 groups than those after 8 weeks of repairing. Both gross specimens and micro-CT 3D reconstruction results showed the ICA/porous magnesium alloy scaffold was the most effective in repairing osteoporotic bone defects. In the ICA/porous magnesium alloy scaffold group, the bone defect was almost completely repaired, leaving only a small defect with good color and smooth surface. The porous magnesium alloy scaffold had the second best repairing effect, and there was new tissue filling in the osteoporotic bone defect. In the control group, there was bone-like tissue on the surface of the osteoporotic bone defect, which was still large and deep, indicating the result was the worst (Fig. 6A and B).

### Histological staining of samples from osteoporotic bone defects repaired with different scaffolds

**Fig. 7.**
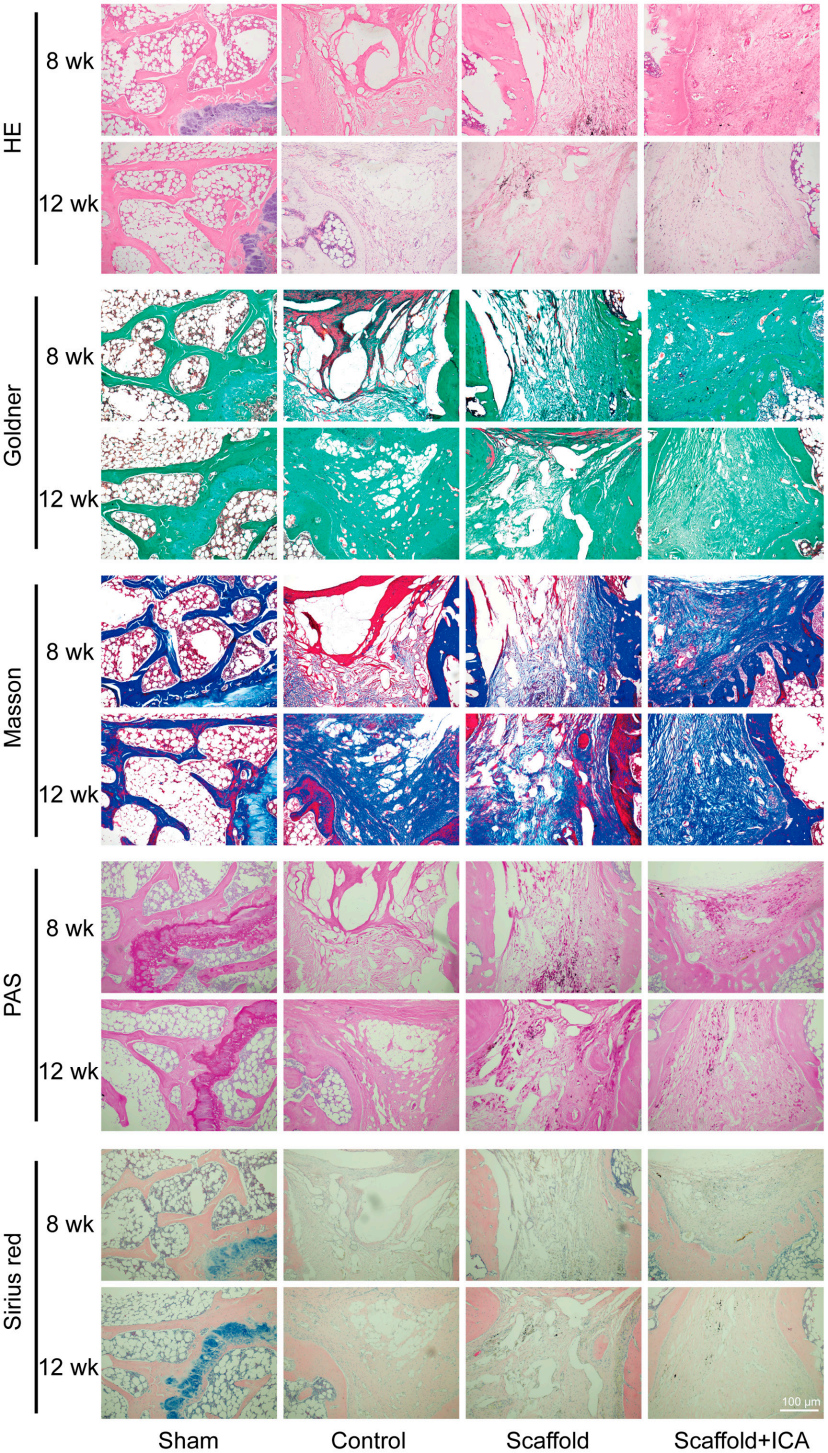
Histological staining of samples from the osteoporotic bone defects repaired with different scaffolds.

HE staining was used to visualize the conventional structure of the layers in the bone defect repaired with different scaffolds in OP rats. Goldner staining was conducted to visualize new bone in the defect. Masson staining and Sirius red staining were carried out to visualize collagen in new bone tissue, and periodic acid-Schiff staining was used to visualize the sugar content in new bone tissue. Compared with that in the other 2 groups, the new bone in the ICA/porous magnesium alloy scaffold group after 8 weeks of repairing was denser and more homogeneous, which was mainly uniformly structured green mineralized bone with more collagen fiber and polysaccharide content. In the porous magnesium alloy scaffold group, new bone generated was more sparsely arranged, and there was a smaller area of green mineralized bone and a smaller area stained with collagen fibers and polysaccharide components. In contrast, a large cavity was still visible in the defect in the control group, with the smallest area of all materials and the worst repair results. Twelve weeks after repairing, the areas of mineralized bone, collagen fibers, and polysaccharide components in the ICA/porous magnesium alloy scaffold group did not differ significantly from those 8 weeks after repairing. For both the porous magnesium scaffold group and the control group, the overall repairing results 12 weeks after repairing were better than those 8 weeks after repairing. For all 3 groups, the change trends of the restoration results 12 weeks after repairing were similar to those 8 weeks after repairing. It demonstrated that the ICA/porous magnesium alloy scaffold could promote the formation of new bone tissue in the defect (Fig. 7).

### The novel ICA/porous magnesium alloy scaffold promotes the repair of osteoporotic bone defects through stimulating the expression of proteins related to the SIRT1-Wnt/β-catenin signaling pathway

**Fig. 8.**
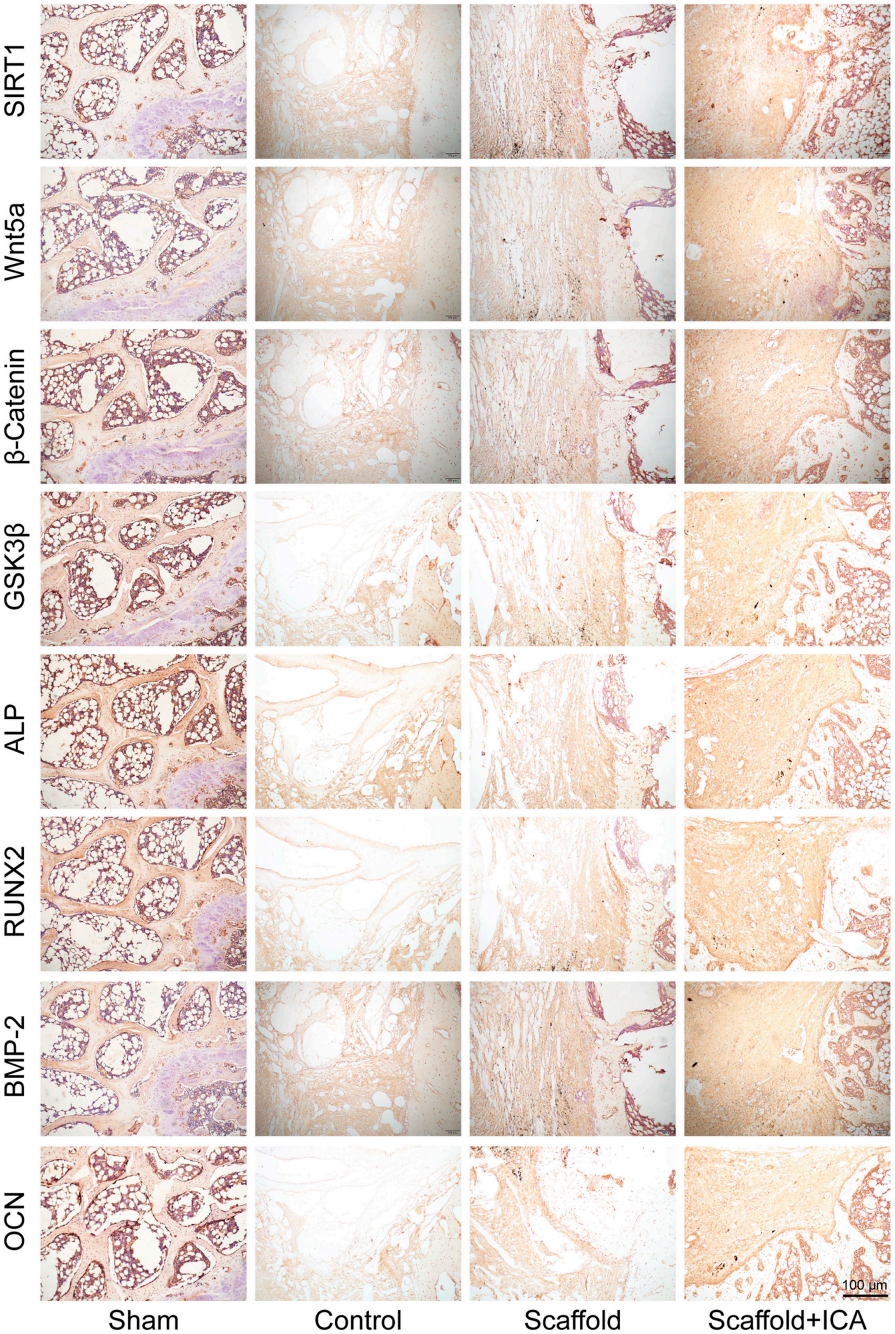
Immunohistochemical staining of samples from the bone defect repaired with different scaffolds.

After 8 weeks of repairing, the expression levels of SIRT1, Wnt5a, β-catenin, GSK3β, ALP, RUNX2, and BMP-2 proteins were significantly higher in the scaffold+ICA group than those in the scaffold group and control group (*P* < 0.05). The expression level of OCN was significantly higher in the scaffold+ICA group than that in the control group (*P* < 0.05). (Fig. 8).

## Discussion

OP is a metabolic disease of the skeleton characterized by impaired bone strength and an increased risk of fractures caused by a variety of factors. OP is also known as the “silent epidemic” [23]. Women with OP over 50 years old are up to 45% more likely to have a fracture of the hip, spine, etc., within 10 years. People over 65 years old have an 87% chance of suffering an osteoporotic fracture in a fall, which predisposes them to osteoporotic bone loss due to reduced bone healing and bone regeneration at this time [24]. However, the treatment modalities already available for osteoporotic bone defects are not effective, and due to its poor self-healing ability, tissue-engineered orthopedic substitutes are often required [25]. Therefore, the novel ICA/porous magnesium alloy scaffold was constructed in the present study. The role of ICA and magnesium ions in promoting osteogenic differentiation of BMSCs and the related mechanism were investigated. The effectiveness and mechanism of the novel ICA/porous magnesium alloy scaffold for repairing osteoporotic bone defects in animals were also studied. The hypothesis that the novel ICA/porous magnesium alloy scaffold could promote the repair of osteoporotic bone defects through the SIRT1-Wnt/β-catenin signaling pathway was finally validated.

ICA is the active ingredient of the Chinese herb Epimedium, capable of inducing actin stress fiber formation via the mitogen-activated protein kinase signaling pathway and strengthening the migration capacity of BMSCs [26]. Factors such as ALP, RUNX2, OCN, and OPN are critical in bone formation [27]. ICA also up-regulates the expression of BMP-2, RUNX2, ALP, and OCN in BMSCs, thereby promoting osteogenic differentiation [28]. In animals, ICA accelerates fracture healing and promotes bone regeneration [29]. In the present study, it was found that after BMSCs were stimulated by ICA, the SIRT1-Wnt/β-catenin signaling pathway was activated, and the expression of SIRT1, β-catenin, p-β-catenin, GSK3β, RUNX2, and BMP-2 proteins was slightly up-regulated in the cells, which ultimately boosted osteogenic differentiation of BMSCs. Therefore, in our experiments, we loaded ICA as a bioactive substance onto the porous magnesium alloy scaffold to construct a novel scaffold material.

Magnesium ions are one of the main macronutrients in the human body, with a content of about 25 g. Sixty percent to 65percent of them are distributed in bones and teeth and about 27% in soft tissue of the body [30,31]. The density of magnesium is 1.74 g/cm^3^, which is close to that of normal human bone tissue (1.8 to 2.0/cm^3^) [6]. Magnesium alloys are therefore more similar to biological bone than other metal materials. The porous magnesium alloy scaffold used in our experiments enhanced the attachment of BMSCs, new osteocytes, and bone tissue and supported new blood vessels while gradually offering magnesium ions to the surrounding tissue as the scaffold degrades. Studies have confirmed that porous magnesium alloy materials show great therapeutic effects in bone defect repairing [32]. Magnesium ions released from the magnesium alloy scaffold implanted in animals during degradation can promote osteogenic differentiation of BMSCs around bone defects and accelerate new bone formation through the Wnt signaling pathway [33]. RNA sequencing of new bone tissue further confirms that magnesium alloy scaffolds accelerate bone healing by activating the Wnt/β-catenin signaling pathway [34]. Our experimental results showed that magnesium ions activated the Wnt/β-catenin signaling pathway, encouraged BMSC proliferation, and greatly increased the mRNA expression of proliferation-related factors SIRT1, Wnt1, Wnt3a, Wnt5a, β-catenin, GSK3β, and GM-CSF proteins compared with that in the absence of stimulators. Besides, magnesium ions also promoted osteogenic differentiation, slightly up-regulated the mRNA expression of osteogenic differentiation-related factors SIRT1, Wnt5a, β-catenin, p-β-catenin, RUNX2, and BMP-2 proteins, and significantly up-regulated the mRNA expression of GSK3β proteins, compared with those in the absence of stimulators. Therefore, we believed that magnesium ions could promote the proliferation and osteogenic differentiation of BMSCs. Compared with that in the control group, the osteoporotic bone defect repaired with the porous magnesium alloy scaffold had a shallower depth and a smaller area, indicating the bone regeneration effect was better.

The Wnt/β-catenin signaling pathway is highly homologous from lower to higher mammals, and in the absence of Wnt ligands, β-catenin is catabolized by degradation complexes in the cytoplasm. When extracellular stimulation occurs, the phosphorylation of β-catenin by the complex is blocked, and the balance of ubiquitinated degradation of β-catenin is disrupted. As β-catenin accumulates to a certain level, it enters the nucleus, affecting the proliferation and osteogenic differentiation of BMSCs [35]. Activation of this pathway up-regulates the expression of osteogenesis-related factors such as ALP, RUNX2, and OCN in osteoblasts and thus enhances osteogenesis [36]. Chen et al. [37] demonstrated that polydatin promoted osteogenic differentiation of human BMSCs through activation of the BMP-2-Wnt/β-catenin signaling pathway, while the osteogenic effect of BMSCs was diminished when the Wnt/β-catenin signaling pathway was specifically blocked by Dickkopf-1. Moreover, SIRT1 is closely related to the Wnt/β-catenin signaling pathway, especially in the process of osteogenic differentiation [38], cell proliferation, cell aging, and apoptosis [39,40]. Nicotinamide mononucleotide promotes bone formation and suppresses adipogenesis by regulating the expression of SIRT1 in aged mice [41]. It has also been shown that the long noncoding RNA TUG/miRNA-204/SIRT1 pathway is directly involved in the osteogenic differentiation of BMSCs [42]. Resveratrol, a specific activator of SIRT1, can also promote bone formation in OP rats by attenuating oxidative stress through the SIRT1/FoxO1 signaling pathway [43]. Liu et al. [44] found that melatonin could reduce oxidative stress damage to cells and enhance cellular osteogenesis through regulating the expression of SIRT1. According to Tao et al. [45], ICA effectively alleviated apoptosis in BMSCs by inhibiting the c-Jun N-terminal kinase/c-Jun signaling pathway and delayed aging by regulating the sirtuin/nuclear factor κB signaling pathway [46]. Magnesium ions also have antiaging and antiapoptosis effects [47].

Through in vitro experiments, the optimal concentrations of 10^−6^ mol/l ICA and 8 mmol/l Mg^2+^ were determined for costimulating the proliferation of BMSCs. It was further found that optimal concentrations of ICA and Mg^2+^ could act synergistically to alleviate cellular senescence and enhance the migration of BMSCs. This finding is consistent with the results achieved by Wang et al. and Liu et al., [48,49] who found that ICA and Mg^2+^ alone had the effect of promoting cell migration. We also noticed that during the proliferation and differentiation of BMSCs, the expression of SIRT1 was correlated with the activation of the Wnt/β-catenin signaling pathway. When the cells were treated with both ICA and Mg^2+^ (at their optimal concentrations), the Wnt/β-catenin signaling pathway was activated, and the expression of SIRT1 was up-regulated compared with that in the control group. The present study showed that 10^−6^ mol/l ICA and 8 mmol/l Mg^2+^ costimulated the proliferation and osteogenic differentiation of BMSCs, and that this process might be achieved through the activation of the SIRT1-Wnt/β-catenin signaling pathway followed by the regulation of osteogenic factor secretion.

In the vivo experiment, we further constructed a novel ICA/porous magnesium alloy scaffold to repair osteoporotic bone defects in rats. It was found that the porous magnesium alloy scaffold promoted the repair of osteoporotic bone defects in rats, and the repair effect improved with the repair time. The novel ICA/porous magnesium alloy scaffold significantly accelerated this process. After 8 and 12 weeks of repairing with the novel ICA/porous magnesium alloy scaffold, the bone defect was smaller in size and shallower in depth, the new trabeculae were denser, and more collagen fibers and polysaccharides were secreted in the defect. As previously mentioned, these changed might be attributed to the fact that the novel ICA/porous magnesium alloy scaffold provides a suitable spatial structure for bone regeneration in the defect and acts as a support for BMSCs, new osteoblasts, and bone tissue, thus facilitating the production of new blood vessels and the secretion of more collagen and polysaccharides. Mechanistic assay suggested that the new ICA/porous magnesium alloy scaffold up-regulated the expression of SIRT1 in osteoporotic bone defects repairing. Based on cellular experimental results, we concluded that the SIRT1-Wnt/β-catenin signaling pathway was activated and the expression of ALP, RUNX2, BMP-2, and OCN proteins were also up-regulated. According to our previous research results, we believe that the activated pathway and up-regulated expression may be because the novel ICA/porous magnesium alloy scaffold acts on BMSCs, osteoblasts, and osteoclasts in the defect and promotes bone formation in a new way. ICA and magnesium ions induce the differentiation of BMSCs into osteoblasts in bone defects and inhibit osteoclastogenesis through hydrogen, another product of magnesium alloy degradation [50]. The interaction of SIRT1 with the Wnt/β-catenin signaling pathway under the combined effect of ICA and magnesium ions promoted the expression of local ALP, RUNX2, BMP-2, and OCN, forming a virtuous circle and accelerating the repair of osteoporotic bone defects in rats.

There are certain limitations to the present study. First, we have not explored the mechanisms by which ICA acts synergistically with magnesium ions to regulate the apoptosis of BMSCs in senescence. Second, we lack direct evidence that up-regulation or down-regulation of the SIRT1 gene or the Wnt/β-catenin signaling pathway affects the repair of osteoporotic bone defects with the novel ICA/porous magnesium alloy scaffold. Finally, we have only conducted preliminary functional and mechanistic experiments at the cellular and small-animal levels, which fall short of practical clinical application. These issues will be the focus of our future research.

In summary, the present study confirms that 10^−6^ mol/l ICA and 8 mmol/l Mg^2+^ can act synergistically to promote the proliferation and osteogenic differentiation of BMSCs in vitro and that this process may be achieved by activating the SIRT1-Wnt/β-catenin signaling pathway. We have successfully constructed a novel ICA/porous magnesium alloy scaffold and found that it can promote the repair of osteoporotic bone defects in rats by activating the SIRT1-Wnt/β-catenin signaling pathway. The results of the present study provide an experimental and theoretical basis for the clinical treatment of osteoporotic bone defects and offer new ideas and strategies to facilitate the repair of osteoporotic bone defects.

## Ethical Approval

The experimental protocol was established, according to the ethical guidelines of the Helsinki Declaration and was approved by the Human Ethics Committee of the Experimental Animal Management Committee of Shenzhen Hospital, Peking University, China (No. 2021-559). Written informed consent was obtained from individual or guardian participants.

## Data Availability

All data generated or analyzed during this study are included in this published article.
