# Supplementary material for: Repair of Osteoporotic Bone Defects in Rats via the Sirtuin 1-Wnt/β-catenin Signaling Pathway by Novel Icariin/Porous Magnesium Alloy Scaffolds
Source: Biomater Res. 2024 Dec 9;28:0090. doi: 10.34133/bmr.0090 (PMC11625907; doi:10.34133/bmr.0090)
Supplement: Supplementary 1 — Figs. S1 and S2 Tables S1 and S2 [file bmr.0090.f1.zip › Supplementary data -BR.docx]

**Supplementary data**

**Supplementary** **Table 1 Primer sequences**

| Gene |  | primer sequence（5′-3′） |
| --- | --- | --- |
| GAPDH |  | Fwd：ATCTGACATGCCGCCTGGAGAA |
|  |  | Rev：ACAACCTGGTCCTCAGTGTAGCC |
| SIRT1 |  | Fwd：CCAAGTTCTATACCCCATGAAGTGC |
|  |  | Rev：CTCGCCACCTAACCTATGACACA |
| Wnt1 |  | Fwd：AACAGTAGTGGCCGATGGTG |
|  |  | Rev：CGGAATTGCCACTTGCACTC |
| Wnt3a |  | Fwd：ACCATGTTCGGGACCTATTCCA |
|  |  | Rev：GCCTGTAGCATCTCGCTTCCA |
| Wnt5a |  | Fwd：ACTTGCACAACAATGAAGCAGGTC |
|  |  | Rev：CAGCCAGCATGTCTTGAGGCTA |
| β-catenin |  | Fwd：CTGATCTCGGACTGGACATTGG |
|  |  | Rev：TCCACCAGAGTGAAAAGAACGG |
| GSK3β |  | Fwd：GACAGACCAATAACGCCGCTTC |
|  |  | Rev：TCCAAACGTGACCAGTGTTGC |
| GM-CSF |  | Fwd：TTCTCCATCCAGAGGCCGACAT |
|  |  | Rev： TGGCTGGCTATCATGGTCAAGG |
| RUNX2 |  | Fwd：GGCCACTTACCACAGAGCTATT |
|  |  | Rev：ATGAGGAATGCGCCCTAAATCA |
| BMP-2 |  | Fwd：GGTCTTTGCACCAAGATGAACAC |
|  |  | Rev：TGGCTTGACGCTTTTCTCGTT |


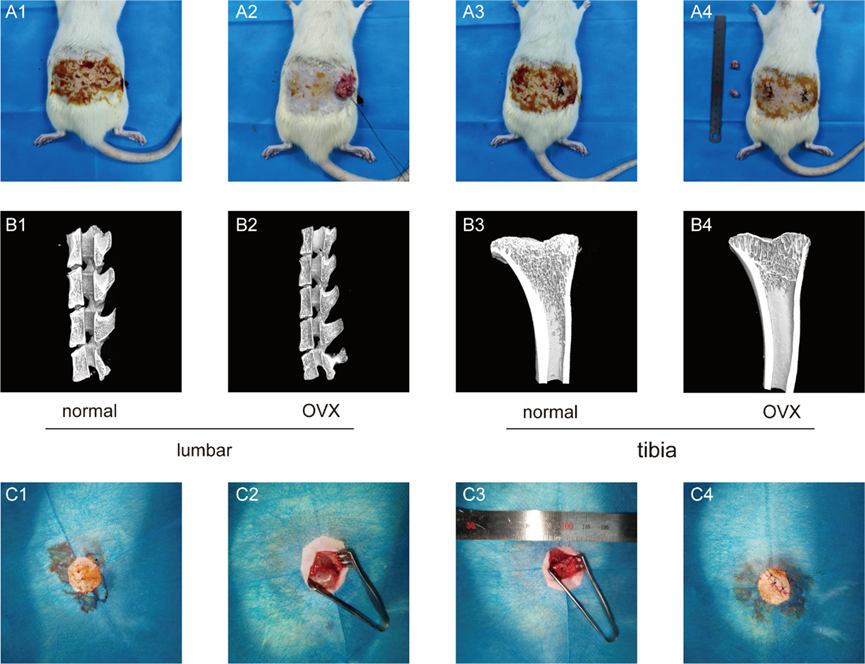


**Figure S1A Establishment of OP models.** A1 shows the surgical area shaved and disinfected, A2 shows the right ovary ligated and removed, A3 shows the right ovary sutured and disinfected, and A4 shows the bilateral ovaries resected. **Figure S1B Validation of OP mouse models.** Micro-CT scans of the lumbar vertebrae, femur and tibia of rats were taken 3 months after OVX. **Figure S1C Modelling of a bone defect in a rat.** C1 shows the right lateral femoral condyle shaved, disinfected and toweled, C2 shows the exposed bone surface of the lateral femoral condyle, C3 shows the creation of a bone defect of 3 mm in diameter and 3 mm in depth, and C4 shows the skin sutured and disinfected after different treatments.

**Supplementary Table 2 Effect of different combinations of ICA and Mg^2+^ on the OD values of BMSCs**

| Groups and days | 1d | 4d |
| --- | --- | --- |
| Control | 1.000 ± 0.000 | 4.790 ± 0.074 |
| 1（10^-8^ mol/L ICA） | 1.072 ± 0.020 | 4.852 ± 0.231 |
| 2（10^-7^ mol/L ICA） | 1.085 ± 0.031 | 4.672 ± 0.726 |
| 3（10^-6^ mol/L ICA） | 1.130 ± 0.024 | 5.252 ± 0.084 |
| 4（10^-5^ mol/L ICA） | 1.087 ± 0.015 | 4.924 ± 0.119 |
| 5（10^-4^ mol/L ICA） | 0.995 ± 0.182 | 4.425 ± 0.122 |
| 6（2 mmol/L Mg^2+^ + 10^-8^ mol/L ICA） | 1.062 ± 0.041 | 4.847 ± 0.122 |
| 7（2 mmol/L Mg^2+^ + 10^-7^ mol/L ICA） | 1.101 ± 0.078 | 4.481 ± 0.077 |
| 8（2 mmol/L Mg^2+^+ 10^-6^ mol/L ICA） | 1.239 ± 0.041** | 4.288 ± 0.296 |
| 9（2 mmol/L Mg^2+^ + 10^-5^ mol/L ICA） | 1.174 ± 0.043** | 4.080 ± 0.343* |
| 10（2 mmol/L Mg^2+^ + 10^-4^ mol/L ICA） | 1.174 ± 0.060** | 4.195 ± 0.315 |
| 11（4 mmol/L Mg^2+^ + 10^-8^ mol/L ICA） | 1.206 ± 0.035** | 6.293 ± 0.178** |
| 12（4 mmol/L Mg^2+^ + 10^-7^ mol/L ICA） | 1.200 ± 0.034** | 6.724 ± 0.341** |
| 13（4 mmol/L Mg^2+^ + 10^-6^ mol/L ICA） | 1.187 ± 0.013** | 6.286 ± 0.187** |
| 14（4 mmol/L Mg^2+^ + 10^-5^ mol/L ICA） | 1.209 ± 0.049** | 6.511 ± 0.538** |
| 15（4 mmol/L Mg^2+^ + 10^-4^ mol/L ICA） | 1.285 ± 0.101** | 5.815 ± 0.037** |
| 16（8 mmol/L Mg^2+^ + 10^-8^ mol/L ICA） | 1.467 ± 0.039** | 6.116 ± 0.260** |
| 17（8 mmol/L Mg^2+^ + 10^-7^ mol/L ICA） | 1.429 ± 0.048** | 6.186 ± 0.113** |
| 18（8 mmol/L Mg^2+^ + 10^-6^ mol/L ICA） | 1.561 ± 0.048** | 6.968 ± 0.406** |
| 19（8 mmol/L Mg^2+^ + 10^-5^ mol/L ICA） | 1.324 ± 0.078** | 6.007 ± 0.143** |
| 20（8 mmol/L Mg^2+^ + 10^-4^ mol/L ICA） | 1.324 ± 0.034 | 5.295 ± 0.245 |
| 21（16 mmol/L Mg^2+^ + 10^-8^ mol/L ICA） | 1.035 ± 0.023 | 4.994 ± 0.040 |
| 22（16 mmol/L Mg^2+^ + 10^-7^ mol/L ICA） | 1.074 ± 0.010 | 5.212 ± 0.078 |
| 23（16 mmol/L Mg^2+^ + 10^-6^ mol/L ICA） | 1.002 ± 0.007 | 4.953 ± 0.036 |
| 24（16 mmol/L Mg^2+^ + 10^-5^ mol/L ICA） | 0.971 ± 0.030 | 4.567 ± 0.071 |
| 25（16 mmol/L Mg^2+^ + 10^-4^ mol/L ICA） | 0.922 ± 0.087 | 4.973 ± 0.364 |
| 26（2 mmol/L Mg^2+^） | 1.006 ± 0.041 | 4.615 ± 0.308 |
| 27（4 mmol/L Mg^2+^） | 0.961 ± 0.020 | 5.145 ± 0.084 |
| 28（8 mmol/L Mg^2+^） | 1.059 ± 0.020 | 5.671 ± 0.089** |
| 29（16 mmol/L Mg^2+^） | 0.924 ± 0.039 | 5.487 ± 0.116* |

Note：* Groups 1-26 vs. Control. ^*^ P＜0.05; ^**^ P＜0.01.


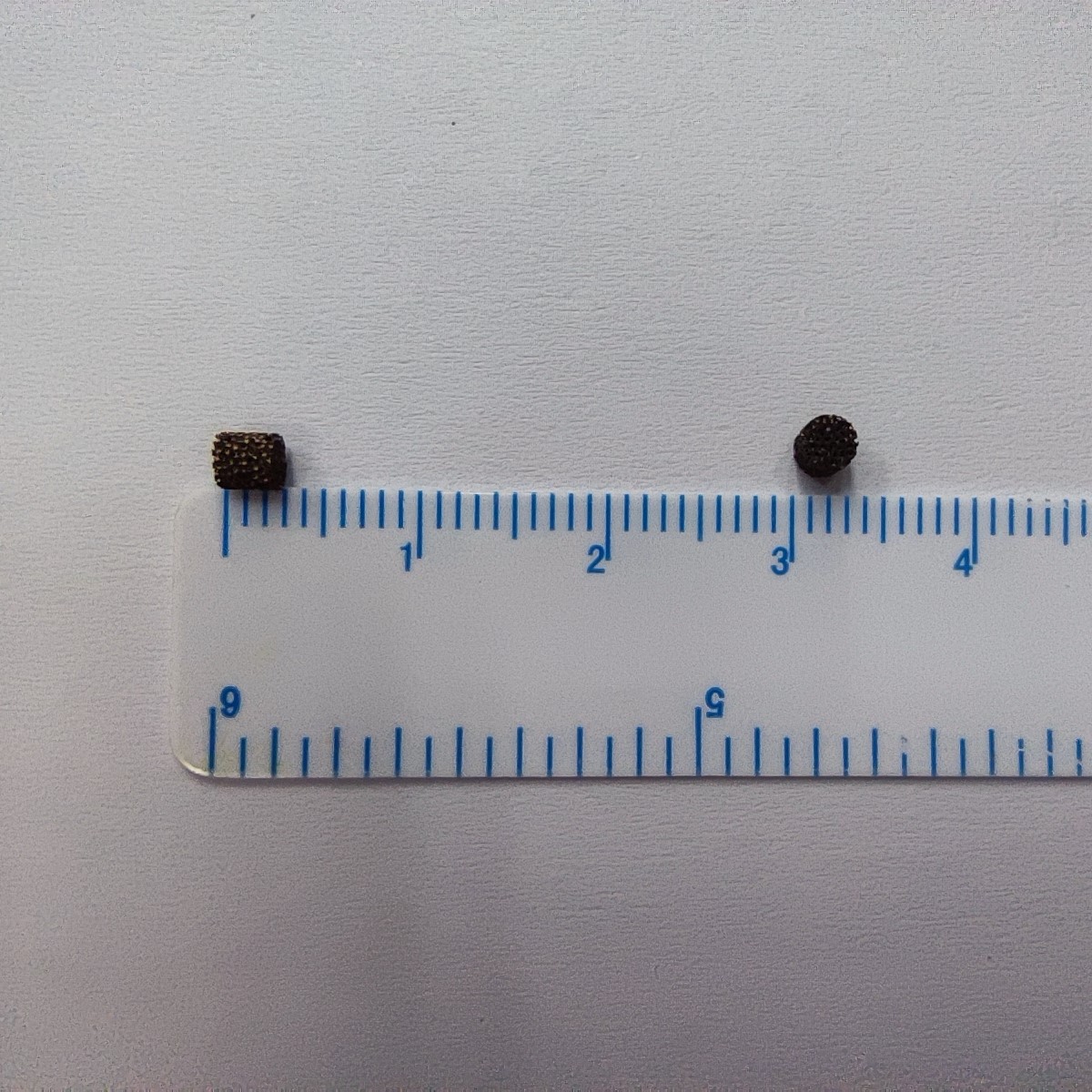


**Figure S2** macroscopic view of the porous magnesium alloy scaffold
