# Supplementary figures and images for: Repair of Osteoporotic Bone Defects in Rats via the Sirtuin 1-Wnt/β-catenin Signaling Pathway by Novel Icariin/Porous Magnesium Alloy Scaffolds
Source: Biomater Res. 2024 Dec 9;28:0090. doi: 10.34133/bmr.0090 (PMC11625907; doi:10.34133/bmr.0090)

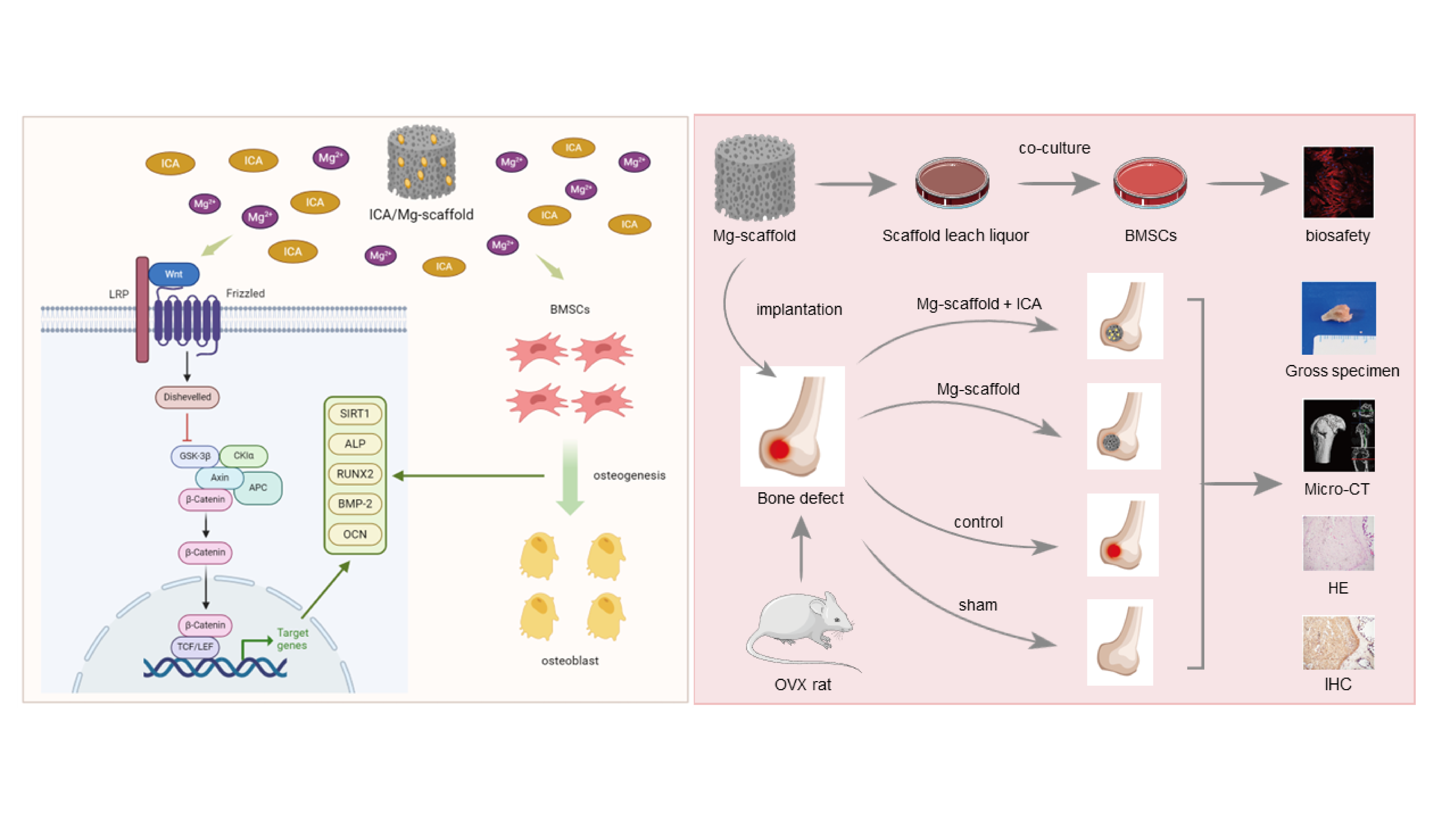

Supplement: Supplementary 1 — Figs. S1 and S2 Tables S1 and S2 [file bmr.0090.f1.zip › TOC-.tif]
